# Supplementary material for: Seminoma and Embryonal Carcinoma Footprints Identified by Analysis of Integrated Genome-Wide Epigenetic and Expression Profiles of Germ Cell Cancer Cell Lines
Source: PLoS One. 2014 Jun 2;9(6):e98330. doi: 10.1371/journal.pone.0098330 (PMC4041891; doi:10.1371/journal.pone.0098330)
Supplement: File S1 — ZIP file containing DMRforPairs output for significant regions. Please start from the html files. (ZIP) [file pone.0098330.s008.zip › figures/23107.pdf]

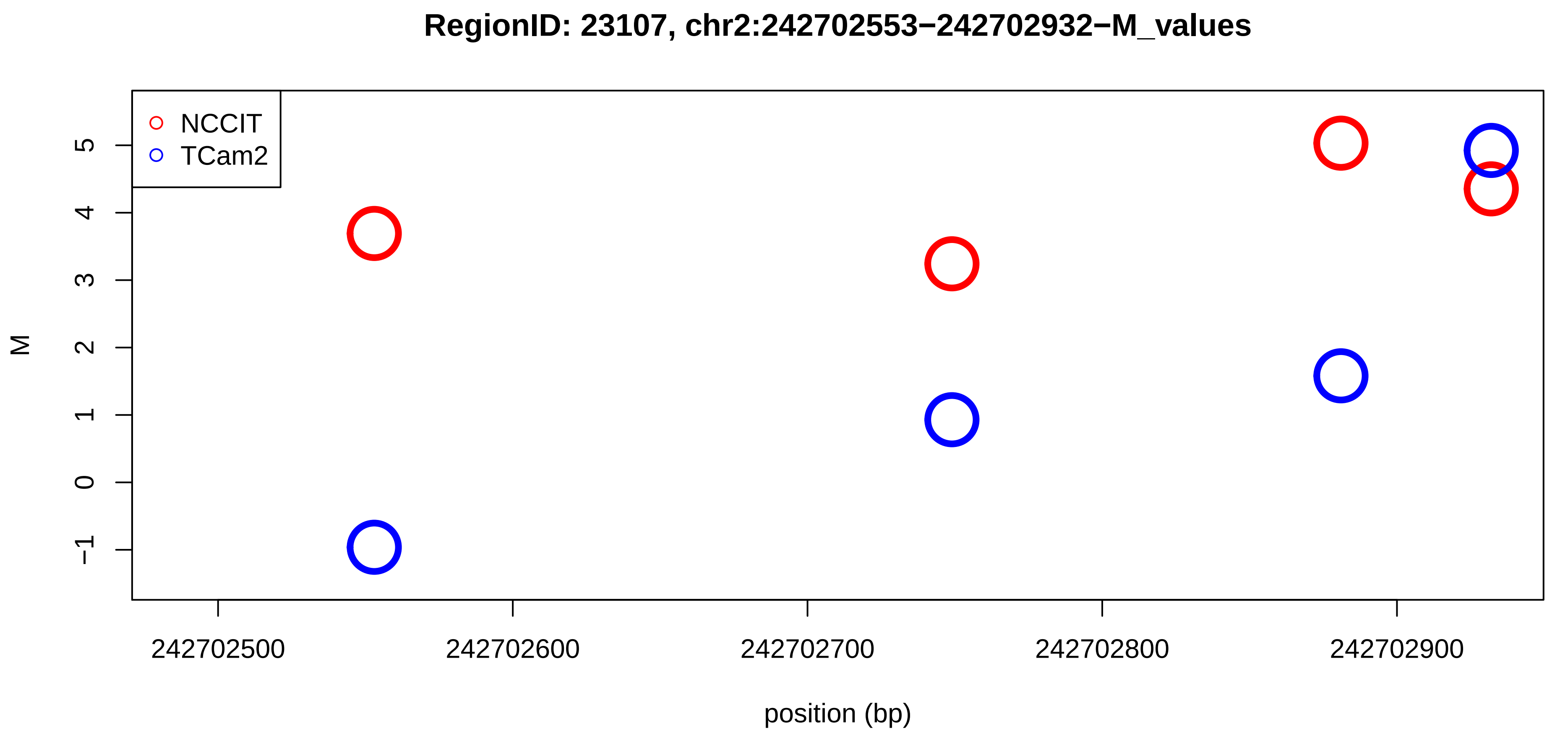

RegionID: 23107, chr2:242702553−242702932−M_values
NCCIT
TCam2
M
−1
0
1
2
3
4
5
242702500
242702600
242702700
242702800
242702900
position (bp)

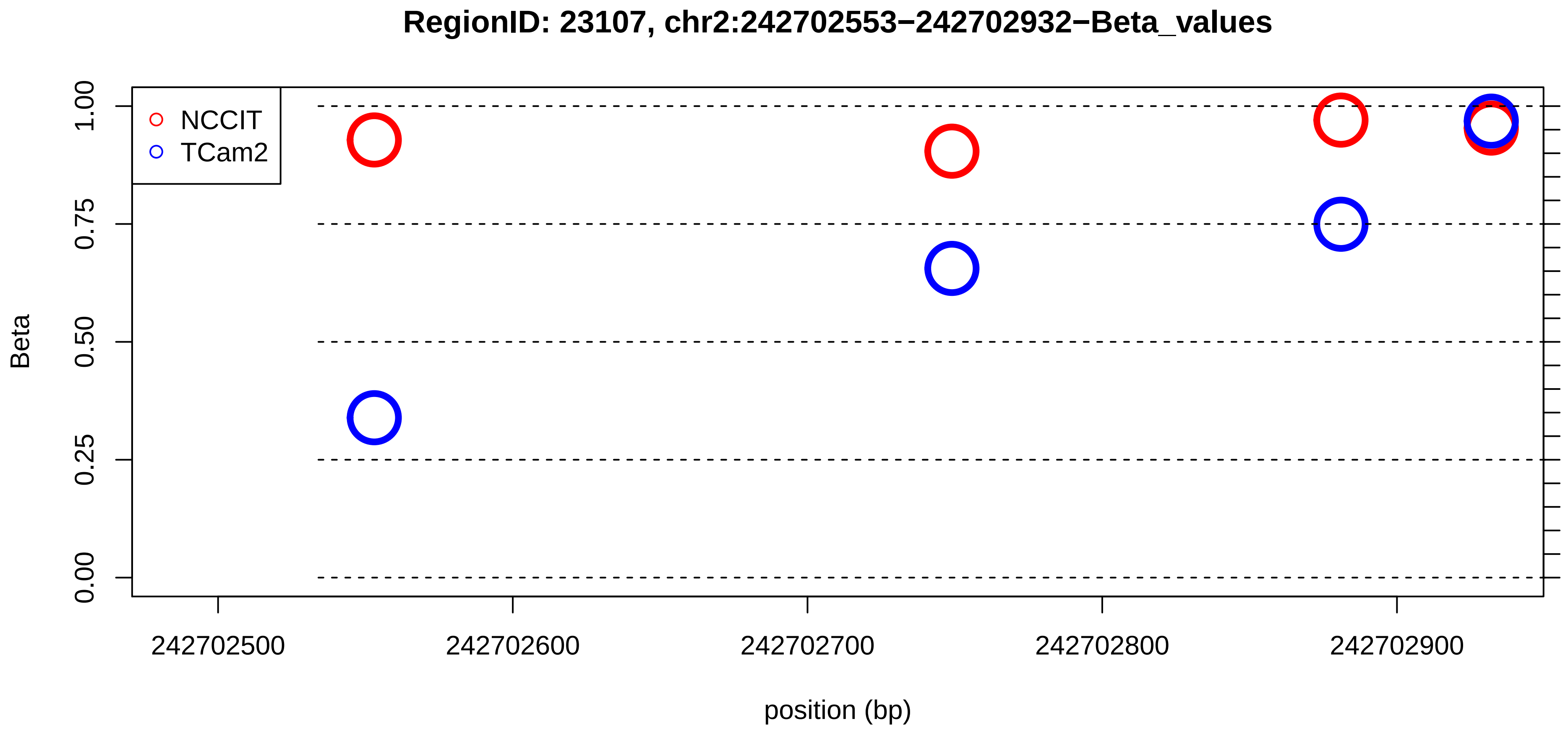

RegionID: 23107, chr2:242702553−242702932−Beta_values
NCCIT
TCam2
Beta
0.00
0.25
0.50
0.75
1.00
242702500
242702600
242702700
242702800
242702900
position (bp)
